# Gastrointestinal complaints in runners are not due to small intestinal bacterial overgrowth

**DOI:** 10.1186/1477-5751-10-8

**Published:** 2011-07-27

**Authors:** Kai Schommer, Dejan Reljic, Peter Bärtsch, Peter Sauer

**Affiliations:** 1 Department of Internal Medicine, The University Hospital Heidelberg, Division of Sports Medicine, Im Neuenheimer Feld 410, 69120 Heidelberg, Germany; 2 Department of Internal Medicine, The University Hospital Heidelberg, Division of Gastroenterology, Im Neuenheimer Feld 410, 69120 Heidelberg, Germany

## Abstract

**Background:**

Gastrointestinal complaints are common among long distance runners. We hypothesised that small intestinal bacterial overgrowth (SIBO) is present in long distance runners frequently afflicted with gastrointestinal complaints.

**Findings:**

Seven long distance runners (5 female, mean age 29.1 years) with gastrointestinal complaints during and immediately after exercise without known gastrointestinal diseases performed Glucose hydrogen breath tests for detection of SIBO one week after a lactose hydrogen breath test checking for lactose intolerance. The most frequent symptoms were diarrhea (5/7, 71%) and flatulence (6/7, 86%). The study was conducted at a laboratory.

In none of the subjects a pathological hydrogen production was observed after the intake of glucose. Only in one athlete a pathological hydrogen production was measured after the intake of lactose suggesting lactose intolerance.

**Conclusions:**

Gastrointestinal disorders in the examined long distance runners were not associated with small intestinal bacterial overgrowth.

## Introduction

Gastrointestinal (GI) disturbances during or immediately after exercise are common among runners [1,2]. 20-50% of long distance runners are affected [3]. Both the upper and lower GI tract are involved. Symptoms are vomiting, nausea, bloating, heartburn and flatulence as well as watery and bloody diarrhea and anal incontinence [4]. The causative mechanisms are not completely understood. The mechanical irritation of the GI tract during running can change intestinal motility [5], additionally exercise causes a reduction of the mesenteric blood flow [6] and both may contribute to the symptoms. Both, a GI dysmotility as well as a reduced mesenteric blood flow are well known risk factors for development of small intestinal bacterial overgrowth (SIBO) [7,8]. Clinical manifestations of SIBO involve the upper and lower GI tract and are similar to the complaints of long distance runners. The gold standard in diagnosing SIBO consists in culture of jejunum aspirate for bacterial counts, but also non-invasive hydrogen breath testing with glucose (GHBT) is well established [9-12]. We hypothesized that due to the high weekly training volume with irritation of GI motility and repeated impairment of the mesenteric perfusion SIBO is present in long distance runners with frequent GI symptoms.

## Methods

Seven long distance runners (5 female, 2 male) were recruited with the help of the headcoach for long distance runners of Baden-Württemberg. Baseline characteristics are given in table 1.

**Table 1:** baseline characteristics

subject	sex	age [years]	body mass index [kg/m^2^]	body fat [%]	training experience [years]	weekly training mileage [kilometers]	training sessions [/week]	running speed at 4 mmol/l lactate threshold [km/h]
**1**	female	25	19.5	14.1	6	120	10	16.6
**2**	male	33	20.6	8.5	9	120	10-12	18.3
**3**	male	27	22.1	13.3	8	60	11	16.8
**4**	female	21	20.8	14.1	6	60	4-6	13.9
**5**	female	33	19.8	15.8	7	100	7	15.6
**6**	female	38	21.8	15.3	17	60	6	13.2
**7**	female	27	22.1	20.7	7	60	6	13.7

We only included otherwise healthy, non-smoking long distance runners with a training experience of ≥ 5 years and a minimum two years lasting, unexplained history of frequent GI complaints (nausea, eructation, heartburn, angina pectoris, vomiting, abdominal cramping, flatulence, diarrhea, or stitch) during or within one hour after running. "Frequent" was defined as at least every other run, and they must have had at least two of the above-mentioned symptoms. By a modified self-assessment questionnaire used in a previous study [13], the following exclusion criteria were assessed: known GI diseases, family history of bowel disease, indication that intake of special food or beverage could explain the GI complaints, intake of antibiotics or proton-pump inhibitors within one month before the study started. Table 2 summarises the GI symptoms reported in the questionnaire. Clinical examination of the abdomen including auscultation and palpation were normal. ECG at rest and during exercise and blood examinations for haemogram, ESR, Aspartate- and Alanine-transaminase, γ-glutamyltransferase, creatinine, urea and ferritine were normal. Body fat composition was determined by 3-point skin fold calipometry [14]. After preexamination, a lactose hydrogen breath test (LHBT) and at least one week later a GHBT on "Wasserstoff-Atemtest" (IFM GmbH, Wettenberg, Germany) were performed after a 12 hour fasting period. These tests were performed in a laboratory of the division of gastroenterology where this examination is routine practice. After two measurements of baseline values for exhaled hydrogen, either 50 g lactose or 75 g glucose (both dissolved in 200 ml of water) was applied and breath samples were analysed for hydrogen every 10 minutes for 3 hours. SIBO is suspected if a clearly recognisable hydrogen peak is present and exhaled hydrogen exceeds 20 parts per million over baseline values in both tests [15,16]. Late hydrogen peaks in the GHBT can be caused by a faster GI transit time for glucose and thus simulate SIBO [9]. Therefore, LHBT was performed as a control in the case of a positive GHBT: SIBO must also result in a positive LHBT [17], but a faster transit time for Glucose does not. Written informed consent was obtained from the subjects, and the study has been approved by the Ethics Commitee of the Medical Faculty of the University of Heidelberg.

**Table 2:** distribution of gastrointestinal symptoms

symptoms	total		during running		after running	
	**n**	**%**	**n**	**%**	**n**	**%**

**nausea**	0	0	0	0	0	0
**eructation**	2	29	1	14	1	14
**heartburn**	2	29	1	14	1	14
**angina pectoris**	0	0	0	0	0	0
**vomiting**	1	14	0	0	1	14
**abdominal cramping**	3	43	3	43	3	43
**flatulence**	6	86	3	43	3	43
**diarrhea**	5	71	3	43	5	71
**stitch**	1	14	0	0	1	14

## Results

**Figure 1:**
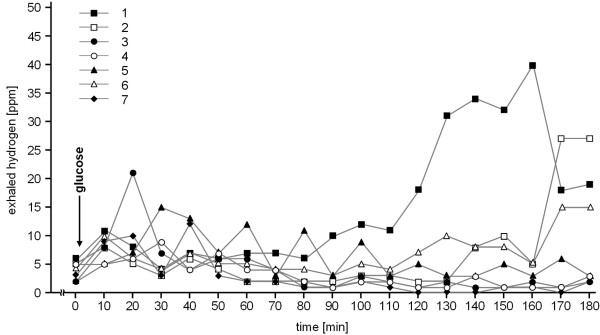
**results of the glucose hydrogen breath test**.

**Figure 2:**
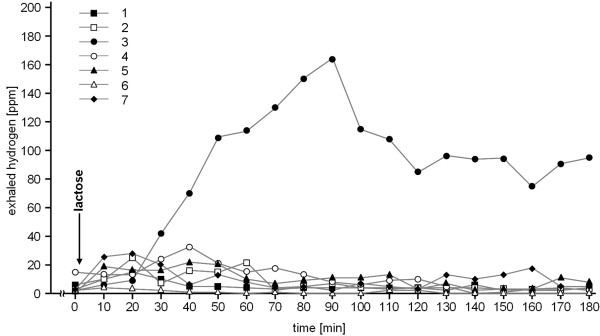
**results of the lactose hydrogen breath test**.

In none of the seven athletes a pathological hydrogen production after application of glucose was observed (Figure 1). In subject 3, a pathological hydrogen production was measured after intake of lactose but not after glucose, suggesting lactose intolerance. Incidentally, this athlete never had any problem after the intake of milk products. In the remaining 6 subjects, LHBT was unremarkable (Figure 2). Subject 4 reported bloody diarrhea after a marathon race two years before. At this time, gastroscopy only revealed some gastric erosions without helicobacter pylori infection whereas colonoscopy was unremarkable.

## Discussion

This study does not provide evidence of SIBO as a common cause accounting for GI problems in long distance runners. All of the investigated runners were frequently afflicted with the usually reported GI symptoms in runners, but none of them showed a pathological GHBT. In the absence of an early peak in this test which could indicate SIBO, the late peak in subjects 1 and 2 in the GHBT is attributable to the passage of the glucose into the colon. This conclusion is supported by the unremarkable LHBT in both runners. The sensitivity and specificity of the GHBT in detecting SIBO was reported to be 62% and 83% [18,19]. With a given prevalence of SIBO in younger adults aged 24 to 59 years of 5-10% [20], the negative predictive value of an unremarkable GHBT is 95-98%. Considering the consistent negative findings in our study we conclude that SIBO is not a common cause for the GI problems of the long distance runner. It appears that the daily duration of the reduced mesenteric blood flow and of the mechanical GI tract concussion does not last long enough in these runners to cause SIBO. The positive LHBT in subject 3 either could be false-positive or indicates a real lactose intolerance. It is reported that lactose maldigesters can usually tolerate small amounts of lactose without symptoms [21]. This could explain why this athlete is asymptomatic except when running. The self-assessment questionnaire did not reveal the intake of lactose-containing food or dietary supplements in connection with running and thus, lactose intolerance is not the reason for the GI symptoms in this athlete.

## Competing interests

The authors declare that they have no competing interests.

## Authors' contributions

KS: conception and design, acquisition, analysis and interpretation of data, drafting the manuscript; DR: acquisition and analysis of data; PB: analysis and interpretation of data, drafting of the manuscript; PS: design, acquisition, analysis and interpretation of data, drafting of the manuscript. All authors read and approved the final manuscript.

## References

[B1] Simons SM Kennedy RG Gastrointestinal problems in runners Curr Sports Med Rep 2004 3 2 112 116 14980141 10.1249/00149619-200404000-00011

[B2] Sanchez LD Corwell B Berkoff D Medical problems of marathon runners Am J Emerg Med 2006 24 5 608 615 10.1016/j.ajem.2006.01.023 16938602

[B3] Peters HP Bos M Seebregts L Akkermans LM van Berge Henegouwen GP Bol E Mosterd WL de Vries WR Gastrointestinal symptoms in long-distance runners, cyclists, and triathletes: prevalence, medication, and etiology Am J Gastroenterol 1999 94 6 1570 1581 10.1111/j.1572-0241.1999.01147.x 10364027

[B4] Riddoch C Trinick T Gastrointestinal disturbances in marathon runners Br J Sports Med 1988 22 2 71 74 10.1136/bjsm.22.2.71 3167507 PMC1478552

[B5] de Oliveira EP Burini RC The impact of physical exercise on the gastrointestinal tract Curr Opin Clin Nutr Metab Care 2009 12 5 533 538 10.1097/MCO.0b013e32832e6776 19535976

[B6] Perko MJ Nielsen HB Skak C Clemmesen JO Schroeder TV Secher NH Mesenteric, coeliac and splanchnic blood flow in humans during exercise J Physiol 1998 513 Pt 3 907 913 9824727 10.1111/j.1469-7793.1998.907ba.x PMC2231328

[B7] Rana SV Bhardwaj SB Small intestinal bacterial overgrowth Scand J Gastroenterol 2008 43 9 1030 1037 10.1080/00365520801947074 18609165

[B8] Gupta A Dhiman RK Kumari S Rana S Agarwal R Duseja A Chawla Y Role of small intestinal bacterial overgrowth and delayed gastrointestinal transit time in cirrhotic patients with minimal hepatic encephalopathy J Hepatol 2010 53 5 849 855 10.1016/j.jhep.2010.05.017 20675008

[B9] Simren M Stotzer PO Use and abuse of hydrogen breath tests Gut 2006 55 3 297 303 10.1136/gut.2005.075127 16474100 PMC1856094

[B10] Stotzer PO Kilander AF Comparison of the 1-gram (14)C-D-xylose breath test and the 50-gram hydrogen glucose breath test for diagnosis of small intestinal bacterial overgrowth Digestion 2000 61 3 165 171 10.1159/000007753 10773721

[B11] Rana SV Sinha SK Sikander A Bhasin DK Singh K Study of small intestinal bacterial overgrowth in North Indian patients with irritable bowel syndrome: a case control study Trop Gastroenterol 2008 29 1 23 25 18564663

[B12] Rana SV Sinha SK Lal S Sikander A Singh K Small intestinal bacterial overgrowth in North Indian patients with celiac disease Trop Gastroenterol 2007 28 4 159 161 18416345

[B13] Halvorsen FA Lyng J Glomsaker T Ritland S Gastrointestinal disturbances in marathon runners Br J Sports Med 1990 24 4 266 268 10.1136/bjsm.24.4.266 2097027 PMC1478906

[B14] Lohman TG Skinfolds and body density and their relation to body fatness: a review Hum Biol 1981 53 2 181 225 7239496

[B15] Kerlin P Wong L Breath hydrogen testing in bacterial overgrowth of the small intestine Gastroenterology 1988 95 4 982 988 3410238 10.1016/0016-5085(88)90173-4

[B16] Metz G Gassull MA Drasar BS Jenkins DJ Blendis LM Breath-hydrogen test for small-intestinal bacterial colonisation Lancet 1976 1 7961 668 669 73641 10.1016/s0140-6736(76)92779-3

[B17] Nucera G Gabrielli M Lupascu A Lauritano EC Santoliquido A Cremonini F Cammarota G Tondi P Pola P Gasbarrini G Abnormal breath tests to lactose, fructose and sorbitol in irritable bowel syndrome may be explained by small intestinal bacterial overgrowth Aliment Pharmacol Ther 2005 21 11 1391 1395 10.1111/j.1365-2036.2005.02493.x 15932370

[B18] Corazza G Sorge M Strocchi A Gasbarrini G Glucose-H2 breath test for small intestine bacterial overgrowth Gastroenterology 1990 98 1 253 254 2293596 10.1016/0016-5085(90)91339-8

[B19] Corazza GR Menozzi MG Strocchi A Rasciti L Vaira D Lecchini R Avanzini P Chezzi C Gasbarrini G The diagnosis of small bowel bacterial overgrowth. Reliability of jejunal culture and inadequacy of breath hydrogen testing Gastroenterology 1990 98 2 302 309 2295385 10.1016/0016-5085(90)90818-l

[B20] Parlesak A Klein B Schecher K Bode JC Bode C Prevalence of small bowel bacterial overgrowth and its association with nutrition intake in nonhospitalized older adults J Am Geriatr Soc 2003 51 6 768 773 10.1046/j.1365-2389.2003.51259.x 12757562

[B21] Suarez FL Savaiano DA Levitt MD A comparison of symptoms after the consumption of milk or lactose-hydrolyzed milk by people with self-reported severe lactose intolerance N Engl J Med 1995 333 1 1 4 10.1056/NEJM199507063330101 7776987

